# Lack of Serological and Molecular Evidence of Duck Tembusu Virus Infection in Ducks from South Korea

**DOI:** 10.3390/vetsci11110564

**Published:** 2024-11-13

**Authors:** Sang-Won Kim, Jong-Yeol Park, Ki-Woong Kim, Cheng-Dong Yu, Feng Hu, Jun-Feng Lv, Yu-Feng Li, Se-Yeoun Cha, Hyung-Kwan Jang, Min Kang, Bai Wei

**Affiliations:** 1 Department of Avian Diseases, College of Veterinary Medicine and Center for Avian Disease, Jeonbuk National University, Iksan 54596, Republic of Korea; sangwonkim@jbnu.ac.kr (S.-W.K.);; 2 Shandong Provincial Key Laboratory of Livestock and Poultry Breeding, Institute of Poultry Science, Shandong Academy of Agricultural Sciences, Jinan 250100, China; 3 Bio Disease Control (BIOD) Co., Ltd., Iksan 54596, Republic of Korea

**Keywords:** duck Tembusu virus, serological, molecular, South Korea

## Abstract

The duck Tembusu virus (DTMUV), an emerging flavivirus, has led to severe neurological disorders and substantial economic losses in the duck industry throughout Asia. Considering South Korea’s increasing duck production and its strategic location along the East Asian–Australasian Flyway, this study aimed to assess the presence of DTMUV in South Korea to evaluate potential risks to the poultry industry. We performed a comprehensive serological survey of 1796 serum samples from broiler and breeder ducks collected between 2011 and 2023, alongside molecular detection tests on 51 duck flocks exhibiting suspected clinical signs of DTMUV infection. The absence of serological and molecular evidence for DTMUV or other flavivirus infections suggests that these viruses have not yet affected South Korean duck populations. These findings underscore the critical need for ongoing surveillance, given the virus’s potential to disrupt agriculture and pose public health risks. The study also emphasizes the importance of maintaining stringent biosecurity measures and conducting further research to monitor and prevent DTMUV transmission, particularly due to the possible role of migratory birds and other vectors in spreading zoonotic diseases.

## Simple Summary

The duck Tembusu virus (DTMUV) is a rapidly emerging virus that causes severe neurological disorders and a significant drop in egg production among ducks, leading to major economic losses in the duck industry across Asia. The virus primarily spreads through infected mosquitoes and close contact between infected and healthy birds. Considering South Korea’s status as a major duck producer and its location along a major migratory bird flyway in Asia, there is a potential risk of DTMUV being introduced and spreading within the country. This study was conducted to investigate the presence of DTMUV in South Korean duck populations and assess the potential threat to the industry. Extensive serological and molecular testing demonstrated no evidence of DTMUV, suggesting that the virus has not yet spread to South Korea. The study highlights the importance of continued surveillance and strong biosecurity measures to prevent the introduction and spread of DTMUV, ensuring the health of the duck population and preventing economic losses.

## 1. Introduction

Viral infections significantly affect the duck breeding industry, leading to substantial economic losses worldwide [1]. The duck Tembusu virus (DTMUV) is an emerging flavivirus that causes a severe neurological disorder and acute egg drop syndrome in ducks and some other avian species, including geese, chickens, sparrows, and pigeons [2,3]. DTMUV infection is primarily characterized by a massive decrease in egg production and severe neurological disorders, associated with almost 100% morbidity and up to 30% mortality [2,3]. Additionally, infected ducks exhibit a variety of symptoms, including ovarian hemorrhage and necrosis, ataxia, diarrhea, and spleen and liver enlargement [2,3]. DTMUV was first identified in Malaysia in 1955 from *Culex tritaeniorhynchus* mosquitoes and caused several outbreaks in China from 2010, resulting in tremendous economic losses in the duck industry [3,4]. Furthermore, DTMUV has also spread to other Asian countries and regions, including Thailand and Taiwan, which led to widespread DTMUV infections in these countries and regions [3,5,6]. Tunterak et al. found that 30.4% of individual ducks and 93.3% of duck flocks demonstrated DTMUV seropositivity since the first case reported in 2013 in Thailand [7]. Considering the recent regional expansion and associated large economic losses in the poultry industry, DTMUV should be regarded as an emerging infectious disease in Asian countries.

DTMUV is a member of the Ntaya virus (NTAV) group belonging to the genus *Flavivirus* of the family *Flaviviridae* [3]. Although DTMUV has mostly been isolated from ducks, it also seems to prefer *Culex* mosquitoes as a vector, as do other flaviviruses, such as the West Nile virus (WNV) and Japanese encephalitis virus (JEV) [8]. Furthermore, the Culex mosquito plays an important role in the dissemination of DTMUV to the bird population [9]. Free-range and migratory wild birds may also be hosts for DTMUV and spread it like they do other viruses belonging to the family *Flaviviridae* [10]. DTMUV may also infect mammals, and experimental mice infected by this virus were found to exhibit neurological symptoms, leading to death [11]. Moreover, high seroprevalence (71.9%) of DTMUV and DTMUV viral RNA has been detected in humans raising ducks in farms affected by known DTMUV outbreaks [12].

South Korea is an important annual wintering habitat for millions of migratory birds using the East Asian–Australasian Flyway, which passes through countries such as China, Malaysia, Thailand, Australia, and Russia [13]. They have been shown to be important contributors to the dispersal of zoonotic pathogens into new areas that are distant from their origins, such as the highly pathogenic avian influenza virus (HPAIV) and JEV [14,15]. In recent years, duck production in South Korea has grown dramatically, and in 2020, it was the seventh highest duck producer in the world (https://www.fao.org/faostat/en/#data/QCL, accessed on 1 March 2024). Considering the lack of information regarding DTMUV infection in ducks in South Korea, this study was conducted to determine the presence of DTMUV in Korea and assess its potential threat to the duck farming industry. We performed a large-scale, cross-sectional serological survey by collecting serum from broiler and breeder ducks in South Korea during the period between 2011 and 2023. Additionally, ducks exhibiting suspected clinical signs of DTMUV infection were subjected to molecular detection tests.

## 2. Materials and Methods

### 2.1. Sample Collection

In this retrospective survey to detect DTMUV infection in ducks in South Korea, 1796 serum samples were collected from broiler and breeder ducks from 2011 to 2023 by the Center for Poultry Diseases Control as part of their routine surveillance strategy for management of animal diseases (Table 1, Table 2 and Table 3 and Supplementary Material S1). The serum samples were collected from six provinces, namely Chungbuk, Chungnam, Jeonbuk, Jeonnam, Gyeongbuk, and Gyonggi, in the central and western regions of South Korea, which account for over 90% of the duck population in South Korea [16,17]. The ducks were raised in an integrated farming system, wherein each farm comprised 5000–20,000 ducks. The serum samples were heat-inactivated at 56 °C for 30 min and stored at −70 °C until the time for DTMUV antibody detection.

All animal experiments were conducted following the requirements of the National Association of Laboratory Animal Care and the Ethics Committee of Jeonbuk National University (Approval No: JBNU 2020-0162). Informed consent was obtained from the owners of the duck flocks used in this study.

### 2.2. Serological Assays

The serum samples were screened with a commercial competitive enzyme-linked immunosorbent assay (ELISA) using the ID Screen^®^ West Nile Competition Multi-Species (IDVET, Innovative Diagnostics, Montpellier, France). This test was originally developed for the detection of antibodies against the envelope (E) protein of the West Nile virus and cross reactions with other flaviviruses [18,19]. The ELISA was performed by following the manufacturer’s instructions. The signal-to-noise (S/N) ratio for each serum sample was determined using the following formula: S/N = sample optical density (OD)450/negative control OD450. Samples with S/N ratios ≤ 0.40 were considered positive. The samples that tested positive were obtained from ducks immunized with live duck Tembusu viral disease vaccine (Yebio Bioengineering, Qingdao, China). Furthermore, the presence of antibodies against DTMUV in the duck serum samples was tested using a competitive DTMUV ELISA antibody test kit (Ringpu Biotech Inc., Baoding, China). The result was considered valid if the average OD value of the negative control was ≥0.8 and the average OD value of the positive control was ≤50% of that of the negative control. Samples with percentage inhibition (PI) values < 12.6% were considered negative, and those with PI values ≥ 18.4% were considered positive. Samples with PI values between 12.6% and 18.4% were considered suspicious.

### 2.3. Virus Identification

Brain, bursa, liver, spleen, and/or ovary samples were collected from live broiler and breeder ducks exhibiting clinical signs of infection, including decline in egg production, ataxia, paralysis, and/or diarrhea, as well as from dead ducks. The samples were obtained from 51 duck flocks present in five provinces located in the main duck-raising areas of South Korea. Approximately 5–20 dead or clinically symptomatic live ducks were obtained from each farm (Table 4 and Supplementary Material S2). All the samples were stored at −70 °C until the time for testing. Furthermore, viral RNAs were extracted from tissue suspensions using the Viral Gene-SpinTM Viral DNA/RNA Extraction kit (iNtRON Biotechnology, Daejeon, Republic of Korea), and the samples were quantified using the NanoDrop 2000 spectrophotometer (Thermo Fisher Scientific, Waltham, MA, USA). Viral cDNA was generated from RNA samples using GoScript^TM^ reverse transcriptase (Promega, Madison, WI, USA) by following the manufacturer’s instructions [20]. Flavivirus and DTMUV were detected using primer sets targeting NS3 and polyprotein, respectively (Supplementary Materials S3 and S4) [21]. A live attenuated JEV vaccine (Chengdu Institute of Biological Products Co., Ltd., Chengdu, China) and a live duck Tembusu viral disease vaccine (Yebio Bioengineering, Qingdao, China) were used as positive controls for flavivirus and DTMUV detection using polymerase chain reaction (PCR).

### 2.4. Statistical Analysis

Statistical analysis was performed using the IBM SPSS Statistics for Windows, version 21.0 (IBM Corp., Armonk, NY, USA). The data were analyzed using a one-way analysis of variance (ANOVA). Differences were considered statistically significant when *p* < 0.05, *p* < 0.01, and *p* < 0.001.

## 3. Results

### 3.1. Serological Results

For the unvaccinated ducks, the PI values ranged from 0 to 15.30%, remaining below the positive threshold. In contrast, vaccinated ducks, which served as positive controls in the study, exhibited PI values exceeding 80%, confirming the presence of antibodies against DTMUV. These results clearly demonstrate a distinct difference in PI values between vaccinated and unvaccinated ducks.

In total, 1796 serum samples from both broiler and breeder ducks were analyzed for the presence of DTMUV antibodies using two different ELISA kits: the IDVET West Nile Competition Multi-Species kit and the competitive DTMUV ELISA antibody test kit. The serum samples, collected from 2011 to 2023, represented a broad range of age groups and geographic regions across South Korea, as outlined in Table 1, Table 2 and Table 3. Each sample was tested with both ELISA kits. Based on our results, none of the samples demonstrated seropositivity, with PI values below the threshold of 18.4%. These findings confirm the absence of detectable DTMUV antibodies in the ducks sampled during this study.

### 3.2. Molecular Detection Results

As part of the molecular testing, we amplified 407 bp and 379 bp DNA fragments using primers targeting flaviviruses (NS3) and DTMUV (polyprotein), respectively. To ensure the accuracy of the PCR amplification, we sequenced these DNA fragments. The sequences were then compared with the reference sequences from the vaccine strains. The results confirmed that the amplified 407 bp fragment corresponds accurately to JEV, and the 379 bp fragment matches the expected sequence for DTMUV.

Samples were obtained from 51 duck flocks, including live broiler and breeder ducks, showing clinical signs of infection, such as decline in egg production, ataxia, paralysis, and diarrhea, as well as dead ducks. A total of 102 PCR tests were performed (51 flocks, each tested using two different primer sets). Despite using specific primer sets to detect flaviviruses (NS3) and DTMUV (polyprotein), the PCR analysis did not reveal the presence of these viruses in any of the tissue samples.

## 4. Discussion

DTMUV was first documented in 2010 as an emerging infectious disease in Asian countries. This study represents the first systematic effort to retrospectively investigate DTMUV infection in South Korea over a period of 10 years from 2011, following the outbreak of DTMUV in China. Fortunately, no active or past DTMUV infection was detected in any of the study populations of ducks until 2023.

In this study, to confirm the presence of DTMUV infection, breeder and broiler ducks were selected to investigate the existence of DTMUV in South Korea. Previous studies have shown that ducks of all ages are susceptible to DTMUV, and this virus can be transmitted efficiently among ducks in all age groups [22,23]. Breeder ducks are exposed for prolonged time to various environmental conditions and other animals throughout their lives, increasing their chances of exposure to DTMUV. This, in turn, increases the likelihood of detecting antibodies if DTMUV is present in the environment or wild birds and animals in South Korea. Negative results from serological examinations of the breeder ducks and even in those of age < 64 weeks demonstrated the absence of DTMUV infection in ducks between 2011 and 2023 in South Korea. Additionally, serological testing of samples collected from broiler ducks during the same period yielded identical negative results, which further proves that the ducks in the sampled farms were not exposed to DTMUV. Furthermore, analysis of all the serum samples using the antibody against flaviviruses showed that the ducks were not exposed to past infection not only by DTMUV but also other viruses in the family of flaviviruses. These results conform to those of previous studies. Although flaviviruses have been detected in wild birds, there is less evidence for the presence of flaviviruses in poultry [24,25,26].

In this study, we employed molecular techniques to detect DTMUV antigens in breeder ducks showing neurological symptoms, reduced egg production, ovarian inflammation, and oviduct hemorrhage, as well as in broiler ducks that had succumbed to illness. DTMUV is known to cause systemic infection, manifesting severe clinical symptoms such as egg drop, ataxia, diarrhea, splenomegaly, and significant reproductive damage, often leading to mortality [27,28]. Despite the presence of these severe clinical symptoms in this study population, DTMUV antigens were absent in our samples. Hence, they may be attributed to other pathogens, either indicating primary infections or part of mixed infections. Similar clinical presentations in ducks can arise from infections due to other pathogens, including duck hepatitis virus, avian influenza virus, egg drop syndrome virus, *Mycoplasma* spp., *Riemerella anatipestifer*, and *Escherichia coli* with or without immunosuppressive duck circovirus [29,30,31,32]. This aligns with the result of a previous study, which demonstrated similar clinical outcomes in ducks infected with these pathogenic agents [31]. The overlap in symptomatology between DTMUV and other infections underscores the need for comprehensive diagnostic approaches in poultry health management [31]. The aim of future studies should be to differentiate between the effects of various pathogens in cases of complex clinical presentations, ensuring accurate diagnosis and effective disease control strategies in duck populations.

The absence of DTMUV infection in the duck population in our study may be due to several reasons. One of them may be the high biosecurity measures implemented in integrated management farms in South Korea, which reduces exposure of these ducks to potential DTMUV vectors, unlike those reared in free-grazing systems where they are highly exposed to wild birds and mosquitoes [33]. Previous studies have shown that infection rates in free-grazing ducks are higher than those in ducks reared in farms in Thailand [7]. Moreover, the free-range system is the main mode of rearing ducks in these countries. Further, the positive influence of using vaccines to control DTMUV in neighboring countries, including China, must be noted. Following significant economic losses due to DTMUV infection in China, the country approved the use of several live attenuated and killed DTMUV vaccines from 2016, leading to a reduction in the virus’s prevalence on farms [2,19]. This might have decreased the chance of the virus spreading to the environment and wildlife, further reducing the likelihood of transmission to South Korea.

The role of wild birds in DTMUV transmission remains unclear. Most studies of DTMUV in wild birds were focused on birds, such as pigeons and sparrows, which have close contact with farm animals [2,3]. There are only a few reports of DTMUV in migratory birds, with only one known case of a migratory bird testing positive [10]. This raises questions regarding the widespread presence of DTMUV in migratory bird populations and their role in cross-border transmission. While DTMUV has been reported in countries such as Thailand, Malaysia, and China, no cases of DTMUV infection have been documented in other major duck-producing countries in Asia [2]. Additionally, our results conform to those of previous studies regarding other flaviviruses. However, there is little evidence for the presence of JEV and WNV antibodies in wild birds and their role in transmitting the live virus to domestic animals in Korea, with only one study reporting the detection of WNV in free-living pigeons in Korea [24]. Migratory birds could also carry DTMUV and effectively transmit it to free-range birds in the field. Data regarding the early detection of avian influenza viruses have proved the value of sentinel animals and their usefulness in mirroring the infection status of the wild-bird population [34]. Further study is necessary to continue DTMUV surveillance using sentinel birds and focus on highly susceptible vectors, including mosquitoes [9,10]. Moreover, regular surveillance of sentinel birds can increase the chances of detecting DTMUV circulation in subclinically infected wild water birds, even without increased mortality.

## 5. Conclusions

As with other epornitic arboviruses, DTMUV has taken advantage of increasingly favorable conditions over recent decades to spread across Asia and invade new territories. The first outbreaks in China in 2010 led to a reconsideration of the dangers posed by this virus. The transmission of DTMUV is largely facilitated by the conditions of intensive poultry rearing in farms containing thousands of animals. The serological and molecular findings of the present study conducted in South Korea from 2011 to 2023 provide evidence regarding the current absence of DTMUV and other flaviviruses in the country’s duck population. The prevalence of DTMUV in farm animals and evidence of its presence in wild animals in neighboring countries underscore the need to understand and monitor transmission patterns. Considering the susceptibility of farm animals, including duck and chicken, to this virus, continuous research is crucial to safeguarding this delicate ecosystem in South Korea. Furthermore, potential vectors, including mosquitoes and domestic and wild animals, may facilitate the spread of disease outside the farm, as in the case of other flaviviruses. To manage the challenges posed by this emerging virus, continuous vigilance is necessary to ensure early detection and response to any future outbreaks, which would help maintain South Korea’s status as free from these potentially devastating viral infections.

## Figures and Tables

**Table 1:** Origin of serum samples analyzed for DTMUV antibody testing collected from 2011 to 2023.

Duck Species (Year)	Number of Serum Samples												
	2011	2012	2013	2014	2015	2016	2017	2018	2019	2021	2022	2023	Total
Broiler	108	324	36	357	44	6	30			10	50	40	1005
Breeder	38	89	97	126	177	85	28	21	20		60	50	791
Total	146	413	133	483	221	91	58	21	20	10	110	90	1796

**Table 2:** Geographical locations in South Korea from where duck serum samples were collected for DTMUV antibody testing.

Duck Species (Location)	Number of Serum Samples						
	Chungbuk	Chungnam	Jeonbuk	Jeonnam	Gyeongbuk	Gyonggi	Others
Broiler	415	65	16	54	9	162	194
Breeder	41		40	238		30	332
Total	456	65	56	292	9	192	526

**Table 3:** Distribution of duck serum samples collected for DTMUV antibody testing according to age groups in South Korea.

Duck Species (Age in Weeks)	Broiler			Breeder			
	<1	1–3	3–6	6–24	24–48	48–64	>64
Number of serum samples	230	242	533	324	231	120	116

**Table 4:** Details of samples collected from duck flocks exhibiting clinical signs of infection for DTMUV antigen detection.

Duck Species (Year)	Number of Tested Flocks								
	2013	2014	2015	2016	2017	2019	2021	2022	Total
Broiler	2	1	5	1					9
Breeder	3	14	18	3	1	1	1	1	42
Total	5	15	23	4	1	1	1	1	51

## Data Availability

The data presented in this study are available from the corresponding author on reasonable request.

## Supplementary Materials

The following supporting information can be downloaded at https://www.mdpi.com/article/10.3390/vetsci11110564/s1, Supplementary Material S1. Geographical locations from where duck serum samples and those of ducks suspected of having infection were collected in each sampling site. Supplementary Material S2. Information regarding duck farms studied for DTMUV detection. Supplementary Material S3. Detection of flaviviruses and alignment of primer sequences. Supplementary Material S4. Detection of duck Tembusu virus and alignment of primer sequences.
