# Supplementary material for: Lack of Serological and Molecular Evidence of Duck Tembusu Virus Infection in Ducks from South Korea
Source: Vet Sci. 2024 Nov 13;11(11):564. doi: 10.3390/vetsci11110564 (PMC11599125; doi:10.3390/vetsci11110564)
Supplement: Supplementary file 1 [file vetsci-11-00564-s001.zip › vetsci-3281617-supplementary.pdf]

Supplementary Material S1

Geographical locations of duck sera samples and susceptive duck samples collections in each sampling site.

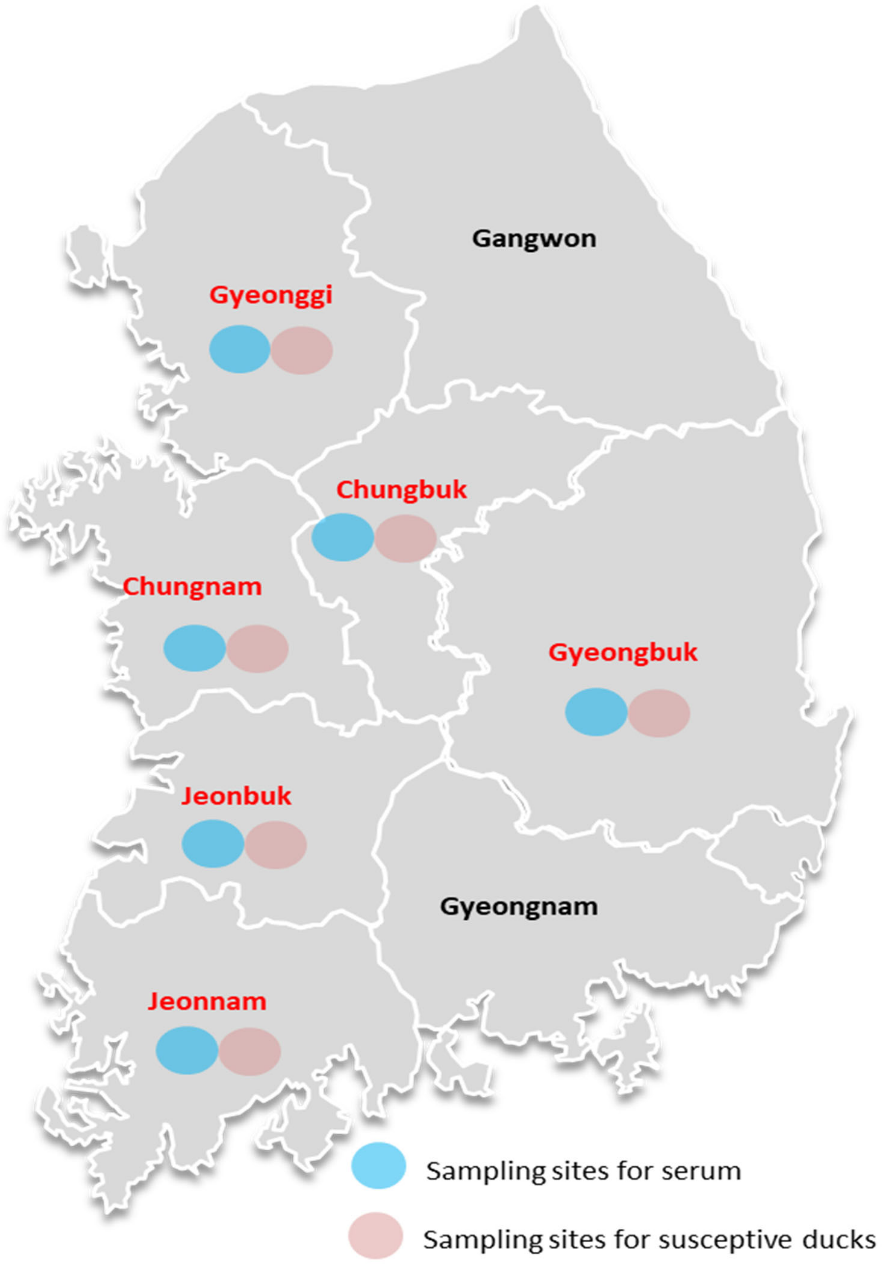

Supplementary Material S2

The information on duck farms for DTMUV detection

No.	Location	Species	Age	Data	Clinical signs	Gross lesions
1	Chungbuk	Breeder	455	2013-10-31	Mortality (3 per day) and egg-laying rate decreased (3-5%)	Ovarian hemorrhage and atrophy, ovarian follicle degeneration, splenic enlargement and hemorrhage, liver degeneration and ascites
2	Jeonnam	Broiler	1	2013-06-04		Splenomegaly with congestion
3	Jeonnam	Broiler	40	2013-06-04	Mortality about 5%	Splenomegaly with congestion
4	Jeonnam	Breeder	82	2013-10-17	Joint swelling (250 cases), lameness, Egg-laying rate decreased (18%)	Splenic enlargement, follicular hemorrhage, salpingitis, liver ischemia, and ascites
5	Jeonnam	Breeder	88	2013-10-23	Neurological symptoms, green stool, joint swelling with limping, and reduced feed intake	Splenomegaly, meningitis, pericarditis, and pericardial edema
6	Jeonnam	Breeder	434	2014-10-10	Egg-laying rate decreased	Intestinal hemorrhage
7	Jeonnam	Breeder	464	2014-10-10	Diarrhea	Intestinal hemorrhage
8	Gyeonggi	Breeder	210	2014-10-13	Egg-laying rate at 44%	Ovarian degeneration
9	Chungbuk	Breeder	98	2014-10-24	Diarrhea	Intestinal hemorrhage
10	Chungbuk	Breeder	203	2014-10-24	Diarrhea	Intestinal hemorrhage
11	Chungbuk	Breeder	224	2014-10-24	Egg-laying rate decreased	Intestinal hemorrhage
12	Chungbuk	Breeder	245	2014-10-24	Egg-laying rate decreased	Follicular hemorrhage
13	Jeonnam	Breeder	462	2014-11-03	Egg-laying rate at 83%. Joint abnormalities	Articular swelling, capsular hemorrhage, and synovitis

14	Jeonnam	Breeder	490	2014-11-03	Diarrhea	Intestinal hemorrhage
15	Jeonbuk	Breeder	182	2014-11-24	Joint abnormalities	Joint edema, arthritis, and hemorrhage, follicular hemorrhage and atrophy
16	Jeonbuk	Breeder	357	2014-11-24	Mortality rate: 10 birds per day<br>Egg-laying rate at 70%, 15% lower than the standard<br>Diarrhea	Arthritis, splenomegaly, ascites, pericardial effusion, hepatomegaly with fibrosis, and ovarian atrophy
17	Jeonbuk	Breeder	413	2014-11-24	Joint abnormalities	Joint swelling, arthritis, follicular atrophy
18	Chungbuk	Breeder	413	2014-11-25	Joint abnormalities<br>Egg-laying rate decreased	Joint swelling, hemorrhage, and inflammation, ascites, hepatic capsular involvement, cirrhosis, and ovarian atrophy
19	Chungbuk	Breeder	277	2014-11-25	Egg-laying rate decreased (1-3%).<br>Abnormal respiratory organ function and green-colored diarrhea	Joint swelling, hemorrhage, and inflammation, ascites, perihepatic effusion, splenomegaly, follicular atrophy and hemorrhage, follicular degeneration, renal enlargement, and uric acid deposition
20	Chungbuk	Broiler	8	2014-12-10	Mortality begins at 7 days of age, with a mortality rate of approximately 9%	Hepatic capsular involvement, renal congestion, tracheal hemorrhage, thymic hemorrhage, pancreatic hemorrhage, and inflammation of the bursa of Fabricius
21	Chungbuk	Breeder	259	2015-01-13	Egg-laying rate decreased (13%)	Focal hepatic necrosis, hair follicle atrophy, salpingitis, and gallbladder enlargement
22	Jeonnam	Breeder	140	2015-01-20	Mortality rate of approximately 1%.<br>Neurological symptoms, including head shaking, depression, cervical dystonia, and tremors	Splenomegaly, salpingitis, arthritis, and cerebral hemorrhage

23	Jeonnam	Breeder	210	2015-01-20	Lameness (300 animals were culled due to joint and lameness symptoms)	Arthritis, splenomegaly, ascites, hepatic degeneration, follicular atrophy.
24	Gangwon	Breeder	-	2015-03-06	Diarrhea	Abnormal eggshell, follicular atresia and hemorrhage, tubal atrophy and hemorrhage, splenic congestion
25	Chungbuk	Broiler	38	2015-04-08	Mortality about 2%	Splenomegaly with congestion
26	Gangwon	Breeder	-	2015-04-21	Diarrhea	Ascites, splenomegaly with congestion, ovarian degeneration, and atrophy
27	Chungbuk	Breeder	105	2015-04-22	Egg-laying rate decreased	Ovarian degeneration, arthritis, salpingitis, bursitis, follicle atrophy
28	Chungnam	Breeder	126	2015-05-21	Neurological symptoms (including uncontrollable wing movements and falling), and arthritis	Hepatic atrophy
29	Gyeongbuk	Broiler	8	2015-06-08	Mortality begins at 6 days of age, with a mortality rate of approximately 27.4%<br>Neurological symptoms	Renal congestion
30	Chungbuk	Broiler	9	2015-06-10	Paralysis followed by death (total of 130 cases)	Renal congestion
31	Gyeonggi	Breeder	304	2015-06-15	Egg-laying rate decreased	Follicular hemorrhage
32	Jeonnam	Breeder	154	2015-06-20	Egg-laying rate decreased	Intestinal hemorrhage
33	Gyeongbuk	Broiler	10	2015-07-06	A total of 700 deaths	Intestinal bloating
34	Chungbuk	Breeder	203	2015-08-01	Diarrhea	Hepatic atrophy, joint hemorrhage, intestinal hemorrhage, and distension
35	Jeonnam	Broiler	39	2015-08-05	Mortality about 2%	Splenomegaly with congestion, intestinal distension and necrosis, and internal hemorrhage

36	Jeonnam	Breeder	48	2015-08-05	Green diarrhea	Splenomegaly
37	Jeonnam	Breeder	232	2015-08-05	Egg-laying rate decreased	Intestinal hemorrhage
38	Chungbuk	Breeder	266	2015-08-12	Mortality (4-5 per day)<br>Egg-laying rate decreased	Hepatic degeneration, arthritis, pancreatic and intestinal hemorrhage, peritonitis, and ovarian degeneration with atrophy
39	Jeonnam	Breeder	109	2015-10-06	Diarrhea	Hepatic capsular involvement
40	Chungbuk	Breeder	175	2015-11-03	Mortality (5 per day)<br>Green diarrhea and joint swelling	Follicular hemorrhage
41	Gyeonggi	Breeder	446	2015-11-03	Egg-laying rate decreased	Follicular hemorrhage
42	Jeonnam	Breeder	293	2015-11-04	Egg-laying rate decreased	Intestinal hemorrhage
43	Jeonnam	Breeder	130	2015-11-06	Green diarrhea	Hepatic hemorrhage with localized necrosis, intestinal distension, hemorrhage in the bursa, and splenomegaly with congestion
44	Chungbuk	Breeder	329	2016-01-12	Egg-laying rate decreased	Hepatic capsular involvement, ascites, splenomegaly, ovarian hemorrhage and atrophy, arthritis, and edema
45	Jeonnam	Breeder	116	2016-01-14	Diarrhea	Hepatic hemorrhage with localized necrosis, intestinal distension, hemorrhage in the bursa, and splenomegaly with congestion
46	Jeonnam	Breeder	172	2016-03-11	Green diarrhea	Hemorrhage and distension in the cecum, arthritis, and hemorrhage
47	Jeonnam	Broiler	19	2016-04-05	A total of 7,000 deaths from 2 days	Renal uric acid deposition
48	Jeonbuk	Breeder	224	2017-02-17	Egg-laying rate has decreased to 86%	Arthritis, follicular hemorrhage, soft-shelled eggs, splenomegaly, airsacculitis

49	Jeonnam	Breeder	315	2019-07-10	Weakness	Mild enteritis, intestinal hemorrhage, ovarian follicular hemorrhage, inadequate follicular development, hepatic ischemia, capsular involvement, and inflammation
50	Jeonnam	Breeder	130	2021-11-06	Green diarrhea	Hepatic hemorrhage with localized necrosis, intestinal distension, hemorrhage in the bursa, and splenomegaly with congestion
51	Gangwon	Breeder	339	2022-06-16	Foot dermatitis, ascites, and periocular exudates	Splenomegaly, intestinal hemorrhage, thymic atrophy, ovarian yolk sac rupture, and underdevelopment of the oviducts

Supplementary Material S3

Flavivirus detection primers

Primer	Nucleotide (5'-3')	Target gene	Size
NS3F	ATGGATGAAGCYCATTTCAC	NS3	407 bp
NS3R	CCAAAGTTGGCYCCCATCTC		

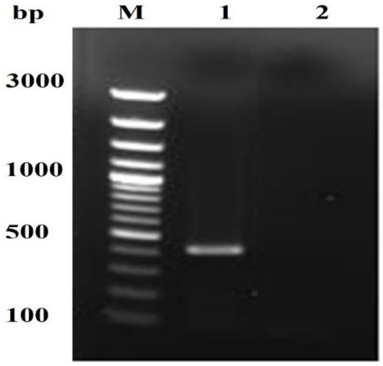

M: 100 bp DNA Marker
Lane 1: JEV
Lane 2: Negative

NS3F:

NS3F_(primer)	ATGGATGAAGCYCATTTCAC
JEV/EF571853.1	ATGGATGAAGCTCATTTCACCGACCCAGCCAGTATAGCCGCACGAGGAT
JEV/U47032.1	ATGGATGAAGCTCATTTCACCGACCCAGCCAGTATAGCCGCACGAGGAT
JEV/HM596272.1	ATGGATGAGGCTCACTTCACTGACCCAGCTAGCATTGCTGCAAGAGGAT
JEV/KM677246.1	ATGGATGAGGCTCACTTCACTGACCCAGCTAGCATTGCTGCAAGAGGAT
JEV/M55506.1	ATGGATGAAGCTCATTTCACCGACCCAGCCAGTATAGCCGCACGAGGAT
DTMUV/MK907880.1	ATGGATGAGGCTCACTTCACAGACCCCGCCAGTATTGCTGCCAGAGGAT
DTMUV/KR061333.1	ATGGATGAGGCTCACTTCACGGACCCCGCCAGTATTGCTGCCAGAGGAT
DTMUV/KY810819.1	ATGGATGAGGCTCACTTCACAGACCCCGCCAGTATTGCTGCCAGAGGAT
DTMUV/KJ489355.1	ATGGATGAGGCTCACTTCACAGACCCCGCCAGTATTGCTGCCAGAGGAT
DTMUV/MN966680.1	ATGGATGAGGCTCACTTCACAGACCCCGCCAGCATTGCTGCCAGAGGAT
ZIKV/MF783072.1	ATGGATGAGGCCCACTTCACAGATCCCTCAAGTATAGCAGCAAGAGGAT
ZIKV/KX377336.1	ATGGATGAGGCCCACTTCACAGATCCCTCAAGTATAGCAGCAAGAGGAT
ZIKV/KU955594.1	ATGGATGAAGCCCACTTCACAGACCCCTCAAGTATAGCTGCAAGAGGAT
ZIKV/PP316140.1	ATGGATGAGGCCCACTTCACAGATCCCTCAAGTATAGCAGCAAGAGGAT
ZIKV/MH882548.1	ATGGATGAGGCCCACTTCACAGATCCCTCAAGTATAGCAGCAAGAGGAT
DV/MH450300.1	ATGGACGAAGCCCACTTTACAGACCCAGCAAGTATAGCAGCTAGAGGAT
DV/MH450307.1	ATGGATGAAGCACATTTCACCGATCCTTCCAGTGTCGCGGCTAGAGGAT
DV/KR919820.1	ATGGACGAAGCACATTTCACTGATCCGGCTAGCATAGCAGCTAGGGGGT

NS3R:

NS3R_(primer)	GAGATGGGRGCCAACTTTGG
JEV/EF571853.1	GAAATGGGGGCCAACTTCGGTGCGAGCAGGGTCATCGACTGTAGGAAGA
JEV/U47032.1	GAAATGGGGGCCAACTTCGGTGCGAGCAGGGTCATCGACTGTAGAAAGA
JEV/HM596272.1	GAGATGGGGGCCAACTTCGGAGCGAGCAGAGTGATTGATTGTAGGAAAA
JEV/KM677246.1	GAGATGGGGGCCAACTTCGGAGCGAGCAGAGTGATTGATTGTAGGAAAA
JEV/M55506.1	GAAATGGGGGCCAACTTCGGTGCGAGCAGGGTCATCGACTGTAGAAAGA
DTMUV/MK907880.1	GAAATGGGAGCGAATTTTGGAGCGCAACGGGTCATAGATAGTCGGAAAT
DTMUV/KR061333.1	GAAATGGGAGCGAACTTTGGAGCGCAACGGGTCATAGATAGTCGGAAGT
DTMUV/KY810819.1	GAAATGGGAGCGAACTTTGGAGCGCAACGGGTCATAGATAGTCGGAAGT
DTMUV/KJ489355.1	GAAATGGGAGCGAACTTTGGAGCGCAACGGGTCATAGATAGTCGGAAGT
DTMUV/MN966680.1	GAAATGGGAGCGAACTTTGGAGCCCAGCGGGTCATAGATAGTCGGAAGT
ZIKV/MF783072.1	GAGATGGGCGCCAACTTTAAAGCTGACCGTGTCATAGATTCCAGGAGAT
ZIKV/KX377336.1	GAGATGGGCGCCAACTTCAAAGCTGACCGGGTCATAGATTCCAGGAGAT
ZIKV/KU955594.1	GAGATGGGCGCCAACTTCAAGGCTGACCGGGTCATAGACTCTAGGAGAT
ZIKV/PP316140.1	GAGATGGGCGCCAACTTTAAAGCTGACCGTGTCATAGATTCCAGGAGAT
ZIKV/MH882548.1	GAGATGGGCGCCAACTTTAAAGCTGACCGTGTCATAGATTCCAGGAGAT
DV/MH450300.1	GAAATGGGTGCCAACTTCAAAGCTGAGAGGGTGATAGACCCCAGACGTT
DV/MH450307.1	GAAATGGGGGCTAACTTTAGAGCTGGGAGAGTGATAGACCCTAGAAGAT
DV/KR919820.1	GAAATGGGAGCGAACTTTAGAGCTGACAGGGTCATAGATCCGAGACGGT

Supplementary Material S4

Duck Tembusu virus detection primers

Primer	Nucleotide (5'-3')	Target gene	Size
CAP3	ATGTCTAACAAAAAACCAGG	Polyprotein	379 bp
CAP4	CAGCCCAGCAACTATCG		

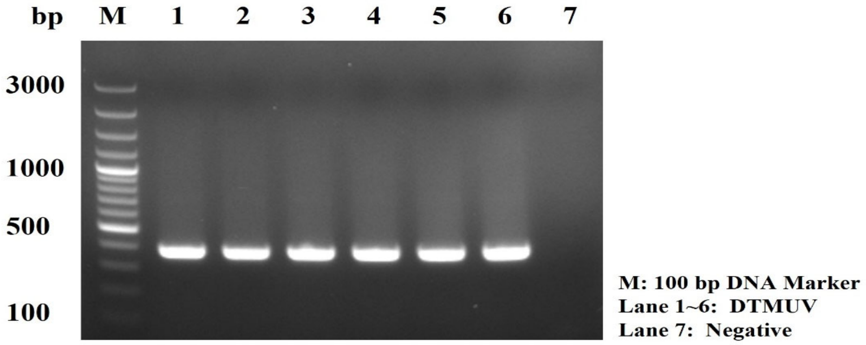

CAP3:

CAP3_(primer)	ATGTCTAACAAAAAACCAGG'''
DTMUV/KT824876.1	ATGTCTAACAAAAAACCAGGAAGACCCGGCTCAGGCCGGGTTGTCAATATGCTAAAGCGCG
DTMUV/KT159713.1	ATGTCTAACAAAAAACCAGGAAGACCCGGCTCAGGCCGGGTTGTCAATATGCTAAAGCGCG
DTMUV/MT447092.1	ATGTCTAACAAAAAACCAGGAAGACCCGGCTCAGGCCGGGTTGTCAATATGCTAAAGCGCG
DTMUV/KM188953.1	ATGTCTAACAAAAAACCAGGAAGACCCGGCTCAGGCCGGGTTGTCAATATGCTAAAGCGCG
DTMUV/KX452096.1	ATGTCTAACAAAAAACCAGGAAGACCCGGCTCAGGCCGGGTTGTCAATATGCTAAAGCGCG
DTMUV/OQ627433.1	ATGTCTAACAAAAAACCAGGAAGACCCGGCTCAGGCCGGGTCGTCAATATGCTAAAGCGCG
DTMUV/OR101160.1	ATGTCTAACAAAAAACCAGGAAGACCCGGCTCAGGCCGGGTTGTCAATATGCTAAAGCGCG
DTMUV/MK907880.1	ATGTCTAACAAAAAACCAGGAAGACCCGGCTCAGGCCGGGTTGTCAATATGCTAAAGCGCG
DTMUV/KY810819.1	ATGTCTAACAAAAAACCAGGAAGACCCGGCTCAGGCCGGGTTGTCAATATGCTAAAGCGCG
DTMUV/KJ489355.1	ATGTCTAACAAAAAACCAGGAAGACCCGGCTCAGGCCGGGTTGTCAATATGCTAAAGCGCG
DTMUV/MN966680.1	ATGTCTAACAAAAAACCAGGAAGACCCGGCTCAGGCCGGGTTGTCAATATGCTAAAGCGCG

CAP4:

CAP4_(primer)	CGATAGTTGCTGGGCTG''
DTMUV/KT824876.1	CGATAGTTGCTGGGCTGAAGCTTGGAAACTATAATGGTAGAGTTTTGGCCACTTTAAATAA
DTMUV/KT159713.1	CGATAGTTGCTGGGCTGAAGCTTGGAAACTATAATGGTAGAGTTTTGGCCACTTTAAATAA
DTMUV/MT447092.1	CGATAATTGCTGGGTTGAAGCTTGGAAACTATAATGGTAGAGTTTTGGCTACTTTAAATAA
DTMUV/KM188953.1	CGATAGTTGCTGGGCTGAAGCTTGGAAATTATAATGGTAGAGTTTTGGCCACTTTAAATAA
DTMUV/KX452096.1	CGATAGTTGCTGGGCTGAAGCTTGGAAACTATAATGGTAGAGTTTTGGCCACTTTAAATAA
DTMUV/OQ627433.1	CGATAGTTGCTGGGCTGAAGCTTGGAAATTACAATGGTAGAGTTTTGGCCACTTTAAACAA
DTMUV/OR101160.1	CGATAGTTGCTGGGCTGAAGCTTGGAAATTACAATGGTAGAGTTTTGGCCACTTTAAATAA
DTMUV/MK907880.1	CGATAGTTGCTGGGCTGAAGCTTGGAAATTATAATGGTAGAGTTTTGGCCACTTTAAATAA
DTMUV/KY810819.1	CGATAGTTGCTGGGCTGAAGCTTGGAAATTATAATGGTAGAGTTTTGGCCACTTTAAATAA
DTMUV/KJ489355.1	CGATAGTTGCTGGGCTGAAGCTTGGAAACTATAATGGTAGAGTTTTGGCCACTTTAAATAA
DTMUV/MN966680.1	CGATAGTTGCTGGGCTGAAGCTTGGAAATTACAATGGTAGAGTTTTGGCCACTTTAAATAA
